# KIR2DL3 and KIR2DL1 show similar impact on licensing of human NK cells

**DOI:** 10.1002/eji.201545757

**Published:** 2015-11-02

**Authors:** Malcolm J. W. Sim, Janet Stowell, Ruhena Sergeant, Daniel M. Altmann, Eric O. Long, Rosemary J. Boyton

**Affiliations:** ^1^ Lung Immunology Group, Infectious Diseases and Immunity, Department of MedicineImperial College London, Hammersmith HospitalLondonUK; ^2^ Laboratory of Immunogenetics, National Institute of Allergy and Infectious DiseasesNational Institutes of HealthRockvilleMDUSA; ^3^ Imperial College Healthcare NHS TrustHammersmith HospitalLondonUK

**Keywords:** Human leukocyte antigen (HLA), Killer immunoglobulin‐like receptor (KIR), Licensing, NK cell (NKC), Rheostat model

## Abstract

Killer cell immunoglobulin‐like receptor/HLA class I (KIR/HLA‐I) combinations are associated with disease risk, implicating functional roles for NK cells (NKCs) or KIR^+^ T cells. KIR/HLA‐I interactions can act through inhibition of NKC activation by target cells and NKC licensing for greater intrinsic responsiveness. We compared licensing conferred by the weaker, HLA‐C group 1/KIR2DL3, and the stronger, HLA‐C group 2/KIR2DL1, inhibitory combinations. The “rheostat model” predicts weaker licensing by HLA‐C1/KIR2DL3 interactions than HLA‐C2/KIR2DL1. We analyzed degranulation in NKC subsets expressing single and multiple receptors for HLA‐I. NKG2A had the strongest licensing impact, while KIR2DL3, KIR2DL1, and KIR3DL1 were weaker, and not significantly different to each other. Presence of one or two matched HLA‐C allotypes did not alter licensing of KIR2DL3^+^ and KIR2DL1^+^ NKC. Coexpression of activating KIR2DS1 disarmed KIR2DL3^+^ and KIR2DL1^+^ NKC to a similar extent. KIR3DL1 and NKG2A combined for more enhanced licensing of double‐positive NKC than the combination of KIR2DL3 and KIR2DL1. Thus, KIR2DL3 and KIR2DL1 have similar capacity to license NKC, suggesting that inhibitory signal strength and amount of available HLA‐C ligands do not correlate with NKC licensing. Altogether, our results show that the basis for disease associations of HLA‐C and KIR2DL likely encompasses factors other than licensing.

## Introduction

NK‐cell (NKC) responses are controlled by signal integration from a diverse array of germ line‐encoded activating and inhibitory receptors 1. The inhibitory killer cell immunoglobulin‐like receptors (KIRs) and the C‐type lectin‐like receptor, CD94‐NKG2A, bind HLA class I (HLA‐I) molecules 2. These receptors utilize phosphorylated tyrosines within immunoreceptor tyrosine‐based inhibition motifs (ITIM) in their cytoplasmic tail to recruit tyrosine phosphatase SHP‐1 upon ligand engagement, leading to dominant inhibitory signals that block NKC activation 3, 4. In addition, inhibitory receptors license NKC, a process whereby functional interactions between inhibitory receptors on NKC and their ligands confer enhanced responses 5, 6, 7. NKC integrate signals from activating receptor combinations, which are subject to negative regulation by dominant inhibitory KIR and NKG2A 8, 9. Summarized by the “rheostat model,” the degree of responsiveness conferred by licensing is tuned according to the number of inhibitory receptor/HLA‐I combinations and the inhibitory signal strength delivered by each receptor/HLA‐I pair 7, 10, 11.

Inhibitory receptor CD94‐NKG2A and its ligand HLA‐E are invariant 12, 13, while KIRs and their HLA‐I ligands are polymorphic 2, 14. KIR binding is determined mainly by conserved epitopes at positions 77 and 80 of HLA‐I 2, 15, 16. KIR2DL2 and 3 bind to the C1 group of HLA‐C alleles while KIR2DL1 binds the C2 group 16, 17, 18, while KIR3DL1 binds to the Bw4 epitope shared by many HLA‐B alleles 2, 19. All HLA‐C alleles contain either the C1 or C2 epitope while only a fraction of HLA‐A and B alleles contain the Bw4 epitope, leading to the idea that HLA‐C function may be principally in NKC regulation 13, 20. KIR/HLA‐I combinations are associated with infection, cancer, autoimmunity, and disorders of pregnancy 2, 12, 21. For example, HLA‐C1 and KIR2DL3 homozygosity associated with early resolution of HCV infection 22. A favored explanation is “strength of inhibition” 23: HLA‐C1 and KIR2DL3 have a weaker interaction than KIR2DL1 and C2, leading to less inhibition and higher NKC responsiveness 2, 24. The “rheostat model” of licensing predicts that weaker interactions, such as KIR2DL3 and HLA‐C1, confer less licensing than stronger ones, such as KIR2DL1 and HLA‐C2. Therefore, it is possible that the protective association of HLA‐C1 and KIR2DL3 with HCV infection is due to weak licensing and lower NK‐cell responsiveness. In theory, the protection observed in KIR2DL3–HLA‐C1 combinations could result from a reduced inhibition of adaptive immunity by NKC 25, 26.

We test this hypothesis by studying licensing conferred by KIR2DL3 and KIR2DL1 with different combinations of HLA‐C1 and HLA‐C2 haplotypes. We find no significant difference in the capacity of KIR2DL3 and KIR2DL1 to confer NKC licensing. KIR2DL3^+^ and KIR2DL1^+^ NK cells (NKCs) were both sensitive to disarming by the activating receptor KIR2DS1 and combined with KIR3DL1, and NKG2A, to confer enhanced licensing of double‐positive (DP) NKC to a similar extent.

## Results and discussion

### NKC licensing by 2DL3 and 2DL1 is not sensitive to HLA‐I ligand number

**Figure 1:**
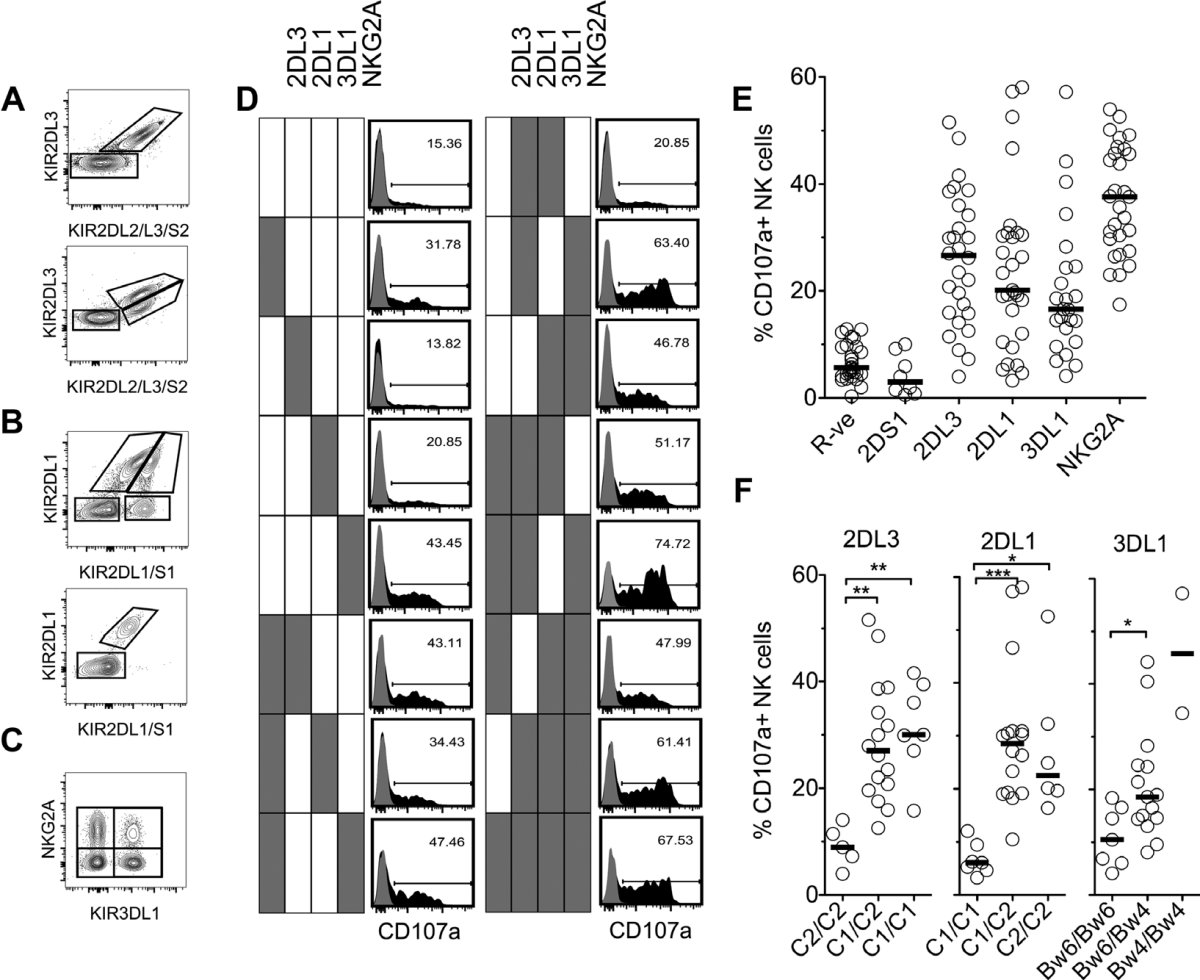
Impact of KIR2DL1 and KIR2DL3 on NKC licensing. (A–C) Representative flow cytometry gating strategy for KIR/NKG2A^+^ NKC from CD56^dim^, CD3^−^ lymphocytes in human PBMCs. (A) Identification of KIR2DL3^+^ and KIR2DL2/S2^+^ NK cells from a KIR2DL3^+^/KIR2DL2^−^/KIR2DS2^−^ genotype donor (*top*) and a KIR2DL3^+^/KIR2DL2^+^/KIR2DS2^+^ genotype donor (*bottom*) with combination of 180 701 and DX27 mAbs. KIRDL2/S2^+^ cells were excluded from analysis as the mAbs do not distinguish KIR2DL2 and KIR2DS2. Data shown are representative of 16/29 (top) and 12/29 (bottom) individuals, respectively. One individual did not carry KIR2DL3. (B) Identification of KIR2DL1^+^, KIR2DS1^+^, and KIR2DL1/S1 double‐positive NKC from a KIR2DL1^+^/KIR2DS1^+^ genotype donor (*top*) and a KIR2DL1^+^/KIR2DS1^−^ genotype donor (*bottom*) with combination of 143 211 and 11PB6 mAbs. Data shown are representative of 8/29 (top) and 21/29 (bottom) individuals, respectively. (C) Identification of NKG2A^+^, KIR3DL1^+^, and NKG2A/KIR3DL1 double‐positive NKC with combination of DX9 and Z199 mAbs. A total of 29/29 individuals are NKG2A+; 24/29 individuals carried 3DL1; two carried 3DL1 high and 3DL1 low alleles. (D) CD107a (H4A3) expression on CD56^dim^, CD3^−^ NKC subsets following K562 stimulation. PBMCs alone (gray‐filled histograms) or mixed with K562 at 5:1 in triplicate wells (black‐filled histograms, one of three wells are shown). Expression of CD107a on all combinations of KIR2DL1^+^, KIR2DL3^+^, KIR3DL1^+^, and NKG2A^+^ NKC from a C1/C1, Bw4/Bw6 KIR2DL2/S2‐KIR2DS1 genotype donor. One triplicate is shown. Filled box indicates receptor positive. (E) Degranulation (CD107a) of NKC subsets expressing single receptors or receptor negative (R‐ve) from 29 healthy volunteers each tested once. Mean CD107a expression from triplicate mixes with K562 minus spontaneous CD107a expression from PBMCs alone. (F) Functional response (CD107a) of KIR2DL3 single positive, KIR2DL1 single positive, and KIR3DL1 single positive NKC to stimulation with K562 cells in donors with 0, 1, or 2 cognate HLA‐I allotypes (from left to right for each KIR). (E and F) Each individual (*n* = 29) was tested once in triplicate degranulation assays with PBMCs alone to quantify spontaneous expression of CD107a. Each circle shown is the mean %CD107a+ minus value from PBMCs alone from a single individual with indicated receptor expression. Black lines represent group medians. Data shown are from 29 independent healthy control individuals. Kruskal–Wallis test with Dunn's multiple comparison test,*p < 0.05; **p < 0.01; ***p < 0.001.

To test whether KIR2DL3 and KIR2DL1 differ in their capacity to license NKC, we recruited 29 healthy volunteers and identified NKC subsets expressing receptors with known roles in licensing: KIR2DL2/S2, KIR2DL3, KIR2DL1, KIR2DS1, KIR3DL1, and NKG2A (Fig. 1A–C and Supporting Information Fig. 1A). NKC subsets expressing no receptors (R‐ve), a single receptor, or up to five receptors were determined in the context of KIR genotypes. The mean percent of each subset is shown in (Supporting Information Fig. 1B) and is in line with previous studies 27, 28. The KIR2DL2/S2 subset was not studied as KIR2DL2 and KIR2DS2 cannot be distinguished serologically. To investigate potential impact of HCMV status on the data in this study, we were able to re‐analyze samples from 19 of our 29 donors; only one had a substantial expansion of CD57^+^, NKG2C^+^ NKCs, indicating most donors were likely not of an HCMV status such as to overtly impact our detected NKC phenotypes 29 (Supporting Information Fig. 2). Our healthy donor ethics did not allow us to directly assess HCMV status.

The responsiveness of each subset was tested by degranulation against K562 targets (Fig. 1D). Receptor negative (R‐ve) NKC and KIR2DS1 single positive cells (KIR2DS1‐SP) were unlicensed and hyporesponsive to activation, whereas NKG2A‐SP, KIR2DL3‐SP, KIR2DL1‐SP, and KIR3DL1‐SP all had enhanced functional responses compared with receptor negative NKC (Fig. 1E). Overall, NKG2A conferred the greatest degree of licensing, while KIR2DL3, KIR2DL1, and KIR3DL1 were weaker (Fig. 1E); some donors had unlicensed populations due to the absence of the corresponding HLA‐I ligand (Fig. 1F). For KIR2DL3 and KIR2DL1, the presence of two HLA‐I ligand alleles (C1/C1 or C2/C2) did not result in greater licensing, in contrast to previous studies addressing this point 30, 31. Licensing conferred by KIR3DL1 was greater in the presence of Bw4/Bw4 as previously described 32, but was not statistically significant since our donor pool contained only two genotypically informative donors (Fig. 1F). Thus, HLA‐C‐specific inhibitory KIRs signal efficiently for licensing.

### KIR2DL3^+^ and KIR2DL1^+^ NKC are sensitive to disarming by KIR2DS1

**Figure 2:**
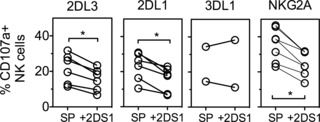
KIR2DL3^+^ and KIR2DL1^+^ NKC are sensitive to disarming by KIR2DS1. Functional response (CD107a) of indicated single receptor positive NKC subsets (SP) to K562 stimulation was compared with subsets co‐expressing the single inhibitory receptor with KIR2DS1. Seven healthy volunteer donors carried KIR2DS1, and all were C1/C2, Bw4^+^. Each circle is from one donor with indicated receptor expression. Connected lines indicate populations from the same donor. Seven healthy control individuals carried KIR2DS1 and HLA‐C2, and were also C1+/Bw4+. Each circle shown is the mean %CD107a+ minus value from PBMCs alone from a single individual with indicated receptor expression. Wilcoxon signed rank test (two‐tailed), *p < 0.05.

We next investigated whether KIR2DL1 and KIR2DL3 differed in relation to other “rheostat model” predictions; “disarming” is a process whereby NKC responsiveness is reduced due to continuous stimulation by activation receptors 33, 34. A previous study showed the activating receptor KIR2DS1, in the presence of an HLA‐C2 ligand, reduced the degree of licensing NKC received from KIR2DL1 and NKG2A, while licensing by KIR2DL3 appeared resistant 30. We compared the ability of KIR2DS1 to “disarm” KIR2DL3^+^, KIR2DL1^+^, KIR3DL1^+^, and NKG2A^+^ NKCs from KIR2DS1^+^ donors carrying C1/C2 and Bw4 haplotypes. Within each donor, NKC co‐expressing KIR2DS1 with a single inhibitory receptor (KIR2DL3, KIR2DL1, or NKG2A) had lower responsiveness by 28, 32, and 30%, respectively, as compared with KIR2DL3‐SP, KIR2DL1‐SP, and NKG2A‐SP NKC (Fig. 2). Thus, NKC licensed by KIR2DL3, KIR2DL1, and NKG2A are susceptible to disarming by KIR2DS1. Compared with previous studies 30, this demonstrates the impact of KIR2DS1 on KIR2DL3 in C1/C2 individuals—that is, disarming of licensing through KIR2DL3 in the presence of KIR2DS1.

### Complementarity of receptor combinations for greater licensing impact

**Figure 3:**
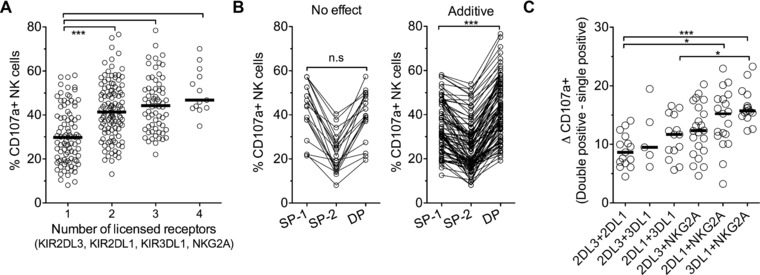
KIR3DL1 and NKG2A combine for greater additive licensing impact than KIR2DL3 and KIR2DL1. (A) Degranulation (CD107a) of NKC subsets expressing 1, 2, 3, or 4 licensing receptors (KIR2DL3, KIR2DL1, KIR3DL1, and NKG2A), from 29 volunteers, previously defined as licensed due to the presence of their respective ligands. (B) Functional responsiveness of double‐positive (DP) NKC were additive (right) or had no effect (left) compared with contributing single positive (SP) subsets (SP‐1 and SP‐2). SP‐1 had greater function of each pair, regardless of receptor identity. No effect was defined as CD107a(DP) ≤ (SP‐1), additive as CD107a(DP) > (SP‐1). Connected lines indicate populations from the same donor. (C) Net degranulation of DP NK subsets defined as additive in Figure 3B stratified by all six combinations of KIR2DL3, KIR2DL1, KIR3DL1, and NKG2A. Degranulation of each SP subset (SP‐1 and SP‐2) was deducted from each DP subset separately, resulting in two values of which the mean was taken and plotted. Black line = median for each group. Each circle shows one donor with indicated receptor expression, horizontal black lines show median values for each group. Data are from all 29 healthy volunteers. Each circle shown is one individual with mean %CD107a+ from triplicate degranulation assays minus value from PBMCs alone from a single individual with indicated receptor expression. Data shown are from 29 independent healthy control individuals. Kruskal–Wallis test with Dunn's multiple comparison test,*p < 0.05; **p < 0.01; ***p < 0.001, n.s: not significant.

In line with the “rheostat model,” NKC expressing one, two, three, and four licensing receptors (KIR2DL3, KIR2DL1, KIR3DL1, and NKG2A) with their respective ligands, showed progressively greater responses (Fig. 3A) 11, 28. DP subsets expressing one licensed and one unlicensed receptor had the same functional responsiveness as the licensed SP subset (Supporting Information Fig. 3). The greatest increment was between NKC expressing one and two receptors; on average SP populations showed 31% CD107a+ degranulation, while DP showed 42% CD107a+ degranulation. However, for a substantial minority (17%) of DP subsets, there was no enhancement and the functional responsiveness was equal to or lower than the greater of the two licensed SP populations (SP‐1; Fig. 3B). These DP subsets across multiple donors included at least one of each of the six combinations of receptors KIR2DL3, KIR2DL1, KIR3DL1, and NKG2A. For the DP subsets with greater function than both SP subsets (additive effect), we calculated the difference (ΔCD107a+) between these DP subsets and their respective single positive populations. As there are two SP populations for each DP, each SP value was deducted from the DP value separately and the mean of the two resulting values was used. The weakest complementarity was between KIR2DL3 and KIR2DL1, with an average of 9% enhanced degranulation, compared with the average of the degranulation observed with KIR2DL3‐SP and KIR2DL1‐SP NKC (Fig. 3C). The receptor combination showing greatest complementarity in promoting licensing was KIR3DL1 and NKG2A, with an average of 17% enhanced degranulation compared with the average of the degranulation observed with KIR3DL1‐SP and NKG2A‐SP NKC. The additive effect of receptor combinations for greater licensing impact increased incrementally, from the lowest with receptors specific for HLA‐C1 and HLA‐C2, to HLA‐C1 or C2 with HLA‐B, HLA‐C1 or C2 with HLA‐E, and the highest with HLA‐B and HLA‐E, suggesting that receptors with more divergent HLA‐I specificities are more complementary for overall licensing impact. This concept of additive education is in keeping with earlier studies [28, 35, 36].

## Concluding remarks

Associations of KIR2DL and HLA‐C with disease have generally been interpreted with respect to “strength of inhibition” 23. For example, the protective combination of HLA‐C1 and KIR2DL3 in clearing HCV infection could be due to the weaker binding of KIR2DL3 to HLA‐C than that of KIR2DL1 or KIR2DL2 22. We tested whether such associations might be explained by differences in impact on NKC licensing. Our data are consistent with the “rheostat model,” demonstrating the disarming role of activating receptor KIR2DS1 and the greater licensing impact of inhibitory receptor combinations. In addition, KIR2DL3 and KIR2DL1 were similar in their capacity to license NKC and to complement KIR3DL1 or NKG2A for enhanced licensing. These results suggest that inhibitory signaling by KIR2DL3 bound to HLA‐C ligands may be as strong as signaling by other KIR–HLA‐C combinations, or that signaling by KIR2DL3 is sufficiently strong to result in similar licensing impact. It is possible that inhibitory KIR receptors may differ in their capacity to recognize changes in peptide repertoire. Indeed, it was recently suggested that KIR2DL2 and KIR2DL3 differ in their peptide selectivity for HLA‐C1 37.

## Materials and methods

### Human donors and ethical approval

All studies were conducted according to the principles expressed in the Declaration of Helsinki. Healthy volunteers were recruited with informed consent with ethical approval through the Imperial College Human Tissue Bank, London (REC number 12/WA/0196).

### PBMC isolation and cell culture

PBMCs were isolated from 20–30 mL peripheral blood by density centrifugation over Histopaque (Sigma, UK) using SepMate (STEMCELL Technologies, France) tubes and cultured in RPMI (GIBCO, Life Technologies, UK), 10% FCS (Labtech, UK). K562 cells were cultured in RPMI, and 10% FCS supplemented with glutamine, penicillin, and streptomycin (GIBCO, Life Technologies).

### KIR and HLA genotyping

Genomic DNA was isolated from peripheral blood by high salt extraction. KIR genotyping was performed using SSP‐PCR (Miltenyi Biotec, UK, 130‐092‐584). HLA‐A, B, and C loci were typed by SSOP PCR.

### Identification of NK subsets

Freshly isolated PBMCs (10^6^)were stained with fluorochrome conjugated mAbs (CD3, CD56, KIR2DL1, KIR2DL3, KIR2DL2/L3/S2, NKG2A, KIR3DL1, and KIR2DL1/S1‐biotin) for 30 min at 4°C, then streptavidin‐APC‐eF780 for 30 min at 4°C. Cells were acquired on an FACs Aria II (BD Biosciences, USA). CD56^dim^, CD3^−^ NKCs were identified from single cells in the lymphocyte gate. Receptor positive populations in the CD56^dim^ gate were first gated for presence of KIR2DL2/S2 and KIR2DL3, then KIR2DL1 and KIR2DS1, then KIR3DL1 and NKG2A (Fig. 1A–C**)**. Sequential gating for the presence or absence of each receptor allowed the identification of NKC subsets expressing up to five receptors and a maximum of 48 different subsets. Frequency of subsets was determined by the number of events in each subset as a proportion of the total events for CD56^dim^, CD3^−^ NKC. Unexpected staining patterns with 11PB6 mAb, likely due to the KIR2DL3*005 allele 38, were found in two donors and excluded from analysis.

### NK‐cell degranulation assay

Freshly isolated PBMCs (10^6^) were mixed with K562 target cells at 5:1 for 2 h in the presence of anti‐CD107a‐BV421 antibody. NKC subsets were identified as above by flow cytometry. Experiments were carried out in triplicate with a no target control to assess spontaneous degranulation (CD107a), the value of which was subtracted from values obtained from cell mixes. Data were analyzed using FlowJo software (v10.07, Treestar Inc., USA).

### Monoclonal antibodies

APC‐KIR3DL1(DX9), Biotin‐KIR2DL1/S1(11PB6), PERCP‐KIR2DL2/L3/S2(DX27) from Miltenyi Biotec. FITC‐KIR2DL3(180701), PE‐KIR2DL1(143211), Alexa‐flour488‐NKG2C(FAB138G) from R&D systems, UK. PE‐Cy7‐NKG2A(Z199) from Beckman Coulter, UK. BV421‐CD107a(H4A3), BV510‐CD3(UCHT1), BV605‐CD56(NCAM16.2) from BD Biosciences, UK. Alexa‐flour647‐CD57(HCD57) from Biolegend, UK.

### Statistical analysis

Statistical analysis was performed using GraphPad Prism V5.0. For comparisons of multiple groups, Kruskal–Wallis test with Dunn's multiple comparison test was used. For comparisons of two groups of paired observations, Wilcoxon signed rank test (two‐tailed) was used. *p* Values of <0.05 were considered significant.

## Conflict of interest

The authors declare that there are no conflicts of interest.

**Abbreviations**
DP: double‐positive
HLA‐I: HLA class I
KIR: killer cell immunoglobulin‐like receptor
NKCs: NK cells

## Supporting information

As a service to our authors and readers, this journal provides supporting information supplied by the authors. Such materials are peer reviewed and may be re‐organized for online delivery, but are not copy‐edited or typeset. Technical support issues arising from supporting information (other than missing files) should be addressed to the authors.

Figure 1. Identification of peripheral blood NKC and NKC subsets expressing different combinations of activating and inhibitory receptors for HLA‐I.
Figure 2. Expression of NKG2C and CD57 on NKC.
Figure 3. Impact of two licensing receptors on NKC responsiveness.
