# Supplementary material for: KIR2DL3 and KIR2DL1 show similar impact on licensing of human NK cells
Source: Eur J Immunol. 2015 Nov 2;46(1):185–91. doi: 10.1002/eji.201545757 (PMC4737201; doi:10.1002/eji.201545757)
Supplement: Supplementary file 1 — Figure 1. Identification of peripheral blood NKC and NKC subsets expressing different combinations of activating and inhibitory receptors for HLA‐I. Figure 2. Expression of NKG2C and CD57 on NKC. Figure 3. Impact of two licensing receptors on NKC responsiveness. [file EJI-46-185-s001.pdf]

European Journal of Immunology

Supporting Information
for
DOI 10.1002/eji.201545757

Malcolm J. W. Sim, Janet Stowell, Ruhena Sergeant, Daniel M. Altmann, Eric O. Long and Rosemary J. Boyton

KIR2DL3 and KIR2DL1 show similar impact on licensing of human NK cells

© 2015 The Authors. *European Journal of Immunology* published by WILEY-VCH Verlag GmbH & Co. KGaA, Weinheim.

European Journal of Immunology

Supporting Information
for
DOI 10.1002/eji.201545757

Malcolm J. W. Sim, Janet Stowell, Ruhena Sergeant, Daniel M. Altmann, Eric O. Long and Rosemary J. Boyton

KIR2DL3 and KIR2DL1 show similar impact on licensing of human NK cells

© 2015 The Authors. *European Journal of Immunology* published by WILEY-VCH Verlag GmbH & Co. KGaA, Weinheim.

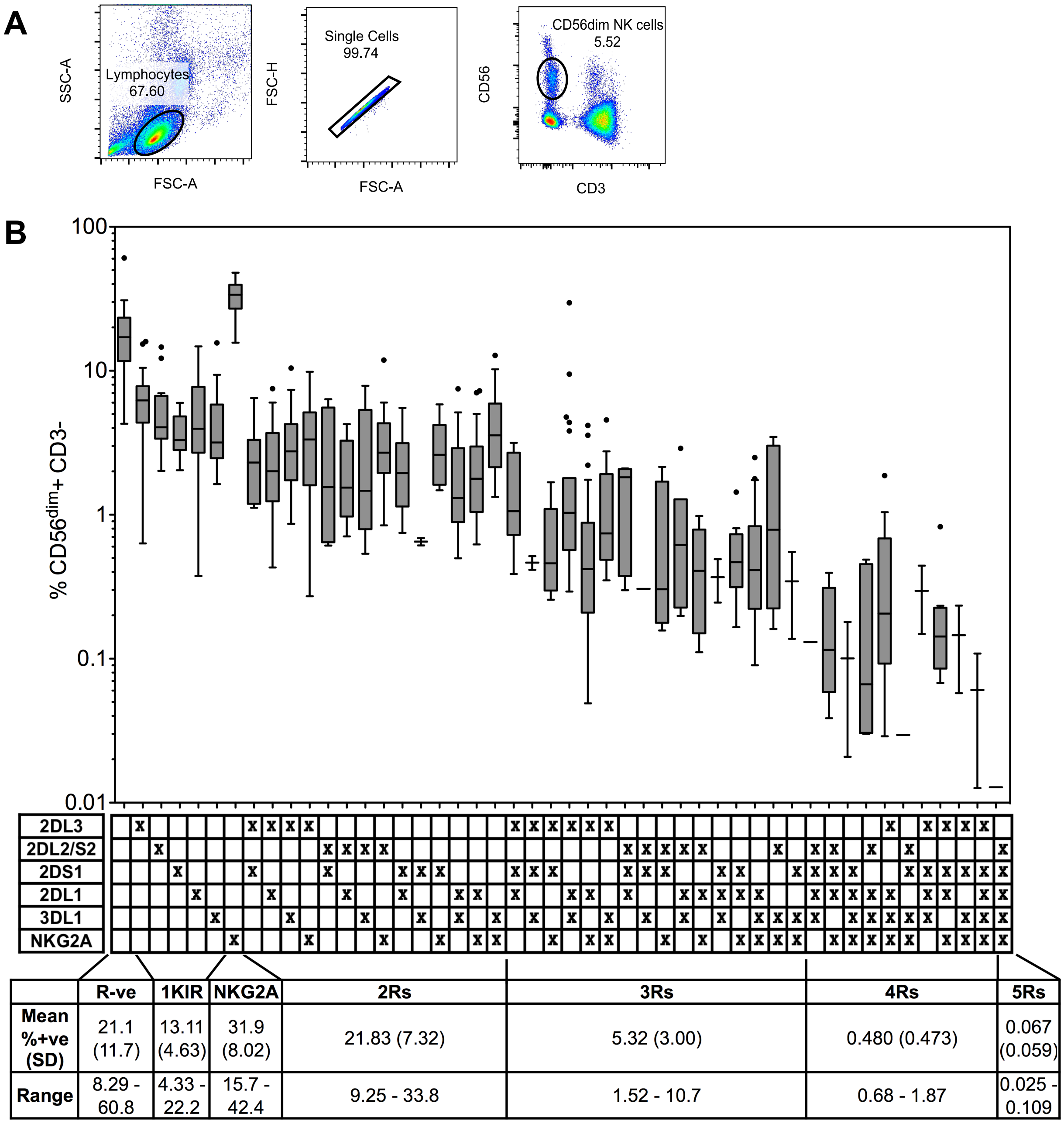

	R-ve	1KIR	NKG2A	2Rs	3Rs	4Rs	5Rs
**Mean %+ve (SD)**	21.1 (11.7)	13.11 (4.63)	31.9 (8.02)	21.83 (7.32)	5.32 (3.00)	0.480 (0.473)	0.067 (0.059)
**Range**	8.29 - 60.8	4.33 - 22.2	15.7 - 42.4	9.25 - 33.8	1.52 - 10.7	0.68 - 1.87	0.025 - 0.109

Supporting Information Figure 1. Identification of peripheral blood NKC and NKC subsets expressing different combinations of activating and inhibitory receptors for HLA-I. (A) Representative flow cytometry gating strategy for identifying CD56^{dim}CD3^{-} NK cells from single cells within the lymphocyte gate. **(B)** The HLA-I receptor repertoire of NKC of 29 healthy volunteers was assessed. CD56^{dim},CD3^{-} lymphocytes were analysed for the expression of multiple receptors and gated for the presence of KIR2DL3, KIR2DL2/S2, KIR2DS1, KIR2DL1, KIR3DL1 and NKG2A. Tukey box plots showing frequency of NKC subpopulations expressing single and combinations of receptors (*top*). The structure of the repertoire is displayed showing the mean, standard deviation and range for NK subsets expressing single KIRs, NKG2A alone, multiple receptors or were receptor negative (*bottom*).

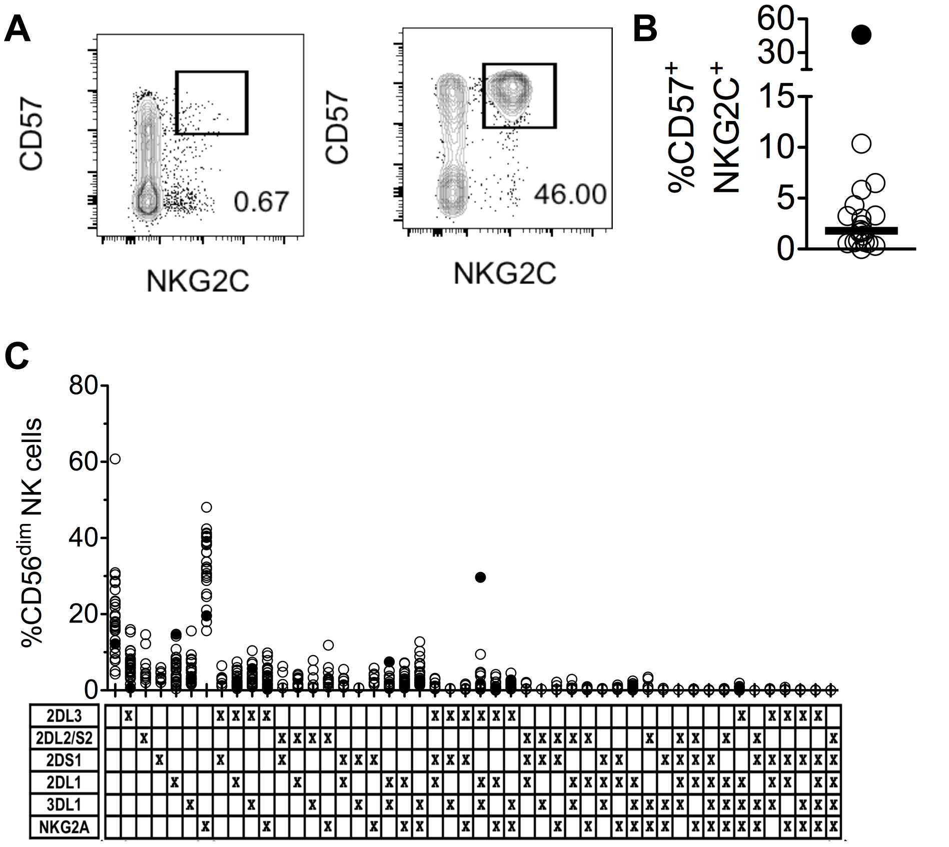

Supporting Information Figure 2. Expression of NKG2C and CD57 on NKC. (A) Representative flow cytometry plots displaying CD57 and NKG2C expression on $CD56^{dim}CD3^{-}$ NK cells from two donors. RHS panel shows the data for donor 9005 **(B)** $CD57^{+}$ and $NKG2C^{+}$ NKCs from 19/29 healthy donors. Horizontal black line shows the median value. **(C)** NKC subpopulations expressing single and combinations of receptors. Each circle is from one individual donor. Black circle shows data for donor 9005 in (B) and (C).

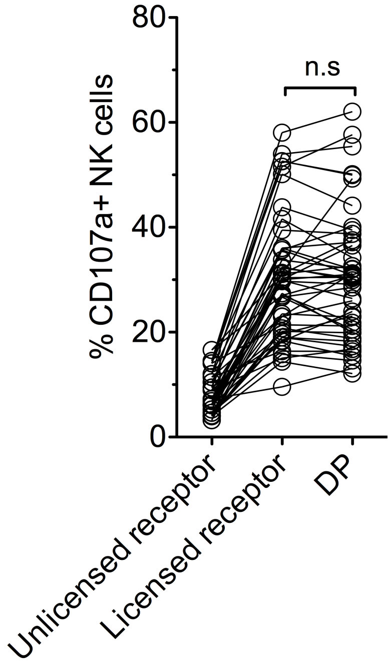

Supporting Information Figure 3. Impact of two licensing receptors on NKC responsiveness. Functional response (CD107a) of double positive (DP) NKC subsets to K562 stimulation with one licensed and one unlicensed receptor. n.s=not statistically significant. Each circle is from one individual donor with the indicated receptor expression (n=29). Connecting lines indicate populations from the same donor.
